# Phenotypic screening of seed retention and histological analysis of the abscission zone in *Festuca pratensis* and *Lolium perenne*

**DOI:** 10.1186/s12870-024-05231-0

**Published:** 2024-06-18

**Authors:** Mareike Kavka, Andreas Balles, Christof Böhm, Klaus J. Dehmer, Christian Fella, Felix Rose, Bernhard Saal, Sabine Schulze, Evelin Willner, Michael Melzer

**Affiliations:** 1 Genebank, Satellite Collections North, Leibniz Institute of Plant Genetics and Crop Plant Research (IPK), Inselstraße 9, 23999 Malchow/Poel, Germany; 2 Development Center X-Ray Technology, Magnetic Resonance and X-Ray Imaging, Fraunhofer IIS, Josef- Martin-Weg 63, 97074 Würzburg, Germany; 3 Saatzucht Steinach GmbH und Co. KG, Wittelsbacherstraße 15, 94377 Steinach, Germany; 4 Physiology and Cell Biology, Structural Cell Biology, Leibniz Institute of Plant Genetics and Crop Plant Research (IPK), Corrensstraße 3, 06466 Gatersleben, Germany; 5 PlantaServ GmbH, Erdinger Straße 82a, 85356 Freising, Germany; 6 Saatzucht Steinach GmbH und Co. KG, Klockower Straße 1, 17219 Ankershagen, Germany

**Keywords:** Seed shattering, Plant genetic resources, Microscopy, Fodder grasses

## Abstract

**Background:**

Seed retention is the basic prerequisite for seed harvest. However, only little breeding progress has been achieved for this trait in the major forage grasses. The aim of this study was to evaluate the potential of plant genetic resources of the important fodder grasses *Festuca pratensis* Huds. and *Lolium perenne* L. as source for seed retention in the breeding process. Furthermore, the morphology of the abscission zone, where shattering occurs, was studied on the cell tissue level in different developmental stages of contrasting accessions.

**Results:**

150 and 286 accessions of *Festuca pratensis* and *Lolium perenne* were screened for seed retention, respectively. Contrasting accessions were selected to be tested in a second year. We found a great variation in seed retention in *Festuca pratensis* and *Lolium perenne*, ranging from 13 to 71% (average: 35%) and 12 to 94% (average: 49%), respectively, in the first year. Seed retention was generally lower in the second year. Cultivars were within the accessions with highest seed retention in *Festuca pratensis*, but had lower seed retention than ecotypes in *Lolium perenne*. Field-shattered seeds had a lower thousand grain weight than retained seeds. Cell layers of the abscission zone appeared already in early seed stages and were nested within each other in accessions with high seed retention, while there were two to three superimposed layers in accessions with low seed retention.

**Conclusions:**

Plant genetic resources of *Lolium perenne* might be a valuable source for breeding varieties with high seed retention. However, simultaneous selection for high seed weight is necessary for developing successful commercial cultivars.

**Supplementary Information:**

The online version contains supplementary material available at 10.1186/s12870-024-05231-0.

## Background

Seed shattering, the detachment of mature seeds, is highly advantageous for seed dispersal of wild plants. Due to adaptations of plants to human utilisation [1], domesticated cereal crops have high seed retention and are therefore easy to harvest and thresh. Some crop weeds have adapted by increasing seed retention in order to be dispersed during harvest of the main crop [2]. On the other side, forage grasses have faced only little artificial selection for seed retention until now [3 and literature cited there]. Main breeding goals are dry matter yield, nutritional quality and resistance to abiotic and biotic stresses [3]. However, seed yield is an essential trait in the breeding process, for commercial seed production and for conservation. Only little breeding progress has been achieved in seed retention, the basic prerequisite for seed yield, in the major forage grasses. Falcinelli [4] could improve seed retention in cocksfoot by repeated backcrossing. Successful attempts using induced radiation mutagenesis were reported by Simon [5, 6] for reed canarygrass, meadow foxtail and meadow fescue (*Festuca pratensis*). With “Cosmolit”, a *Festuca pratensis* cultivar with high seed retention was released in 1993 in Germany and is still noted as such in the descriptive variety list [7].

Seed shattering occurs at the abscission zone (AZ), which is a group of specialised cells [8]. The location of the AZ in the inflorescence of grasses is highly diverse [9, 10]. Grass inflorescences are panicles, racemes or spikes with spikelets containing one or more florets arranged on the rachilla. Most grass species shed a part of the spikelet as diaspore, but also the shedding of whole spikelets or whole inflorescences occurs [11]. Morphology, i.e. cell size, and cell wall composition of the AZ is variable and does not follow phylogenetic relations [10]. In the forage grass *Elymus sibiricus* it has been shown that seed shattering is caused by degradation of the AZ by increased cellulase and polygalacturonase activity which results in a smooth fracture surface on the rachilla in high seed shattering genotypes [12]. Transcriptome profiling confirmed the assumption that increased lignification may play a role in resistance of seed shattering [13]. Yu et al. [10] observed that late shattering and harder to shatter grass species tend to have lignified in comparison to non-lignified AZs. However, between the weedy shattering *Setaria viridis* and the domesticated non-shattering *S. italica* only subtle morphological differences and no lignification of the AZs were found [14].

*Festuca pratensis* and perennial ryegrass (*Lolium perenne*) are the most widely grown grass species for fodder production. Both belonging to the tribe Poeae and the subtribe Loliinae of the grass plant subfamily Pooideae, the inflorescences of *Festuca pratensis* are panicles, whereas the inflorescences of *Lolium perenne* are spikes with unbranched spikelets. In both species, one spikelet consists of several fertile florets. The AZ of *Festuca pratensis* [15] and *Lolium perenne* [16, 17] is located across the rachilla at the base of each floret.

Seed shattering depends on physiological, genetic and environmental factors and the degree of seed shattering differs within species [18]. For *Lolium perenne*, seed retention variability was found between and within different cultivars and ecotypes, but no morphological differences were found in the AZs of contrasting genotypes [16]. Seed retention in *Lolium multiflorum* ranged from 46 to 95% in 56 North American weedy populations under controlled conditions, with diverse intrapopulation ranges [19], indicating breeding potential for this trait. Genetic resources may serve as source for seed retention in fodder grasses. In a recent study [20], seed yield components and seed retention have been evaluated in a genetically diverse set of 21 accessions and lines of *Lolium perenne*. Seed retention ranged from 27% to almost 79%.

In the present study, we used plant genetic resources and cultivars to study seed retention in the important fodder grasses *Festuca pratensis* and *Lolium perenne*. The main aim was to identify genetic resources that can be used in breeding programs to increase seed retention. Therefore, our objectives were (I) to study the variation between and within genebank material compared to cultivars under varying conditions, (II) to correlate seed retention with important characteristics for fodder crop cultivars and (III) to characterise the AZ of in seed retention contrasting genotypes histologically. We show that there is a wide range of degrees of seed retention within both species and that the degree depends on the morphology of the AZ.

## Materials and methods

### Plant material

Seeds (throughout the manuscript, we will refer to the one-seeded grass fruit as seed) of *Festuca pratensis* and *Lolium perenne* from genebank material and from breeding populations and current cultivars of the breeding company Saatzucht Steinach were used for a field experiment in Malchow/Poel in the north of Germany. The plant material of *Festuca pratensis* consisted of 145 ecotypes and landraces from different European countries, maintained at the German federal Ex situ Gene Bank of the Leibniz Institute of Plant Genetics and Crop Plant Research (IPK). It was complemented with two cultivars and three breeding populations of Saatzucht Steinach. The material of *Lolium perenne* consisted of 283 ecotype accessions from different European countries. They originate from different European genebanks and were studied before within the GrassLandscape project (e.g [21]). In addition, twelve cultivars and five breeding populations of Saatzucht Steinach were included. Please refer to the additional file 1 for a list of all used accessions with additional information.

For *Festuca pratensis*, a full-sib family of a cross between Cosmonaut (seed retention of 76% in 2016) and Senu (seed retention of 16% in 2016) comprising seventy-nine F1 individuals was tested for seed retention in two consecutive years in field experiments in Steinach in the south of Germany.

### Field experiments

In Malchow, seeds were sown into small pots in July 2017. Thirty randomly selected young plants were transferred to the field in two replicated blocks in September 2017 (54°00’31"N 11°27’25"E, Poel Island, Germany). Within each block, the 15 plants of every accession were positioned in plots in three rows of five plants (50 cm between rows, 40 cm between plants). Their neighbouring accessions were chosen on the basis of similar maturity dates.

In 2018 and 2019, vigour after winter, biomass, leaf rust (all with scorings from 1 – low to 9 – high), and heading date (in days after April 1^st^) were rated visually per plot. Leaf rust, aphids’ infestations, and empty inflorescences were noted on harvested spikes.

In Steinach, the seeds of the full-sib family of *Festuca pratensis* were sown into small pots and then planted with a distance of 60 × 60 cm between plants without clonal replicates in autumn 2017 (48°57’28’’N 12°36’23’’E, Steinach, Germany).

### Measurement of seed retention

For measurements of seed retention in Malchow, five plants per accession were used in every block in 2018. For 2019, accessions with high and low seed retention, and, additionally, cultivars and accessions in different maturity groups were selected for measurement based on the results of 2018. This resulted in a selection of 17 *Festuca* accessions and 32 *Lolium* accessions, of which 15 plants per block were analysed in 2019. In Steinach, every plant was studied in every year.

About ten tillers of one plant were placed into a closed bag (Crispac, 305 mm x 450 mm, 0.5 mm perforation) after flowering and bound to a stick. Surrounding tillers were cut down. When ripening was completed, tillers were cut and counted. The seeds were assorted to one of three fractions: the seeds already loose in the bag (= field-shattered), seeds that shattered after treatment with an agitation machine (custom built, Jacobs, Halle/Saale, Germany; 20 s with an amplitude of 64 mm and a speed of 150 ms, power: 70%; in 2018 not for *Lolium* in Malchow), and seeds that had to be hand-stripped from the inflorescence. The glumes were still attached to the seeds in all fractions. Empty seeds were removed as well as possible. The fractions were weighed separately.

Thousand grain weight (TGW) of eight selected accessions of *Lolium* was determined in 2018 and 2019. If possible, 50 seeds of the fractions of field-shattered and hand-stripped seeds (ten plants in 2018, 30 plants in 2019) were weighed and their TGW was extrapolated.

### Data analysis

Data of *Festuca* and *Lolium* from Malchow and of the two years were analysed separately. Data of plants with potential seed loss due to damaged bags, plants with poor pollination and all data with less than or equal to 0.5 g total harvested seeds were discarded. Additionally, accessions with less than 5 plants left in total were not considered in the analyses. As indicated in Additional File 1, in 2018, 53 plants (3.5%) within 42 *Festuca* accessions, 14 complete *Lolium* accessions and additional 319 plants (11.2%) within 152 *Lolium* accessions were discarded from the analysis. This resulted in 1447 plants of 150 accessions for *Festuca* in 2018 and 430 plants of 17 accessions in 2019, and 2541 plants of 286 *Lolium* accessions in 2018 and 862 plants of 32 accessions in 2019. If not noted otherwise, calculation of seed retention was based on loose seeds in the field. Data of single plants from Steinach were omitted when loss due to damaged bags was possible.

Statistical analyses were performed using R (version 4.2.1 [22]). A Linear mixed effect model with accession, year, fraction and interactions between accession and year and accession and fraction as fixed effect and block as random effect was fitted for TGW data and an ANOVA performed using the packages “lme4” [23] and “lmerTest” [24]. For correlations, Pearson’s product moment correlation coefficients were estimated. In case of seed retention, the average of all measured plants per accession was used, in case of ratings, the average of the two plots in the field. The coefficient of variation for 2018 seed retention data was calculated per accession as the ratio of the standard deviation to the arithmetic mean of each accession. All figures were created using base R or “ggplot2” [25].

### Microscopy

#### Digital microscopy

**Fig. 1:**
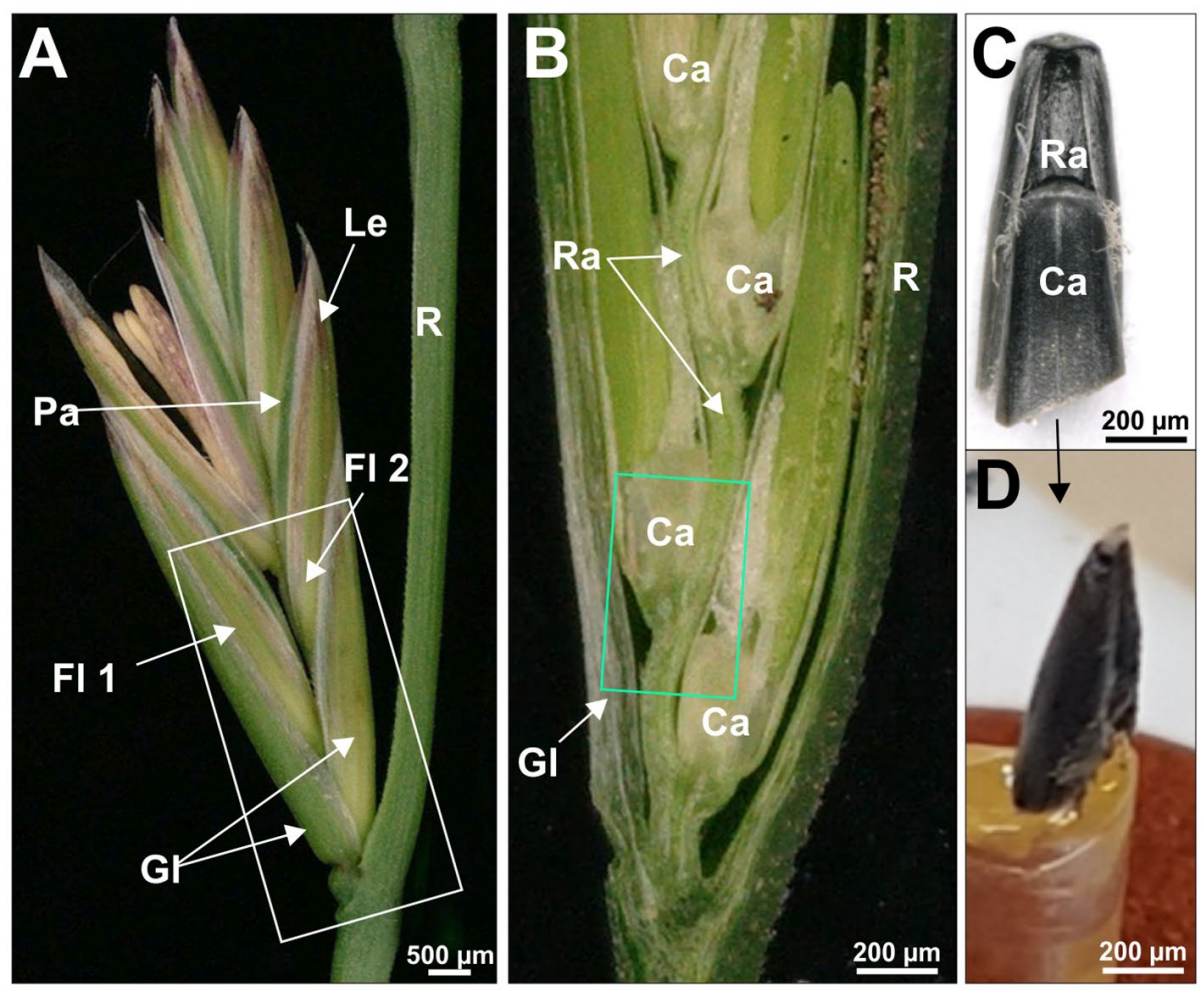
Preparation of the transition zone of developing inflorescences of *Lolium perenne* for microscopy. Image of a complete spikelet (**A**), and close up image of a spikelet cut open in the middle (**B**). Sample for X-Ray microscopy after critical point drying (**C**) and mounted on sample holder for X-ray analysis (**D**). Green boxes indicate tissue of the second floret used for sample preparation for histology and X-ray microscopy. Ca, caryopsis; Fl, floret; Gl, glume; Le, lemma; Pa, Palea; R, rachis; Ra, rachilla

For analysis of the general morphology of the transition area from the rachis to the developing caryopsis, plants with high and low seed retention of various genotypes of *Festuca pratensis*, *Lolium perenne* and *Lolium multiflorum* at different developmental stages were used: before earing, earing, flowering, milk stage, dough stage and maturity. To analyse the morphology and the grade of lignification of the transition area from the rachis to the developing caryopsis, spikelets and dissected florets of contrasting genotypes of selected accessions were imaged (Fig. 1) using a Keyence VHX-5000 digital microscope (Keyence Deutschland GmbH, Neu-Isenburg, Germany). Prior to imaging frontally from above and from the side, glumes of the florets were dissected. In addition, individual florets were cut longitudinally centrally and imaged (Fig. 1B). For staining of lignin, dissected tissue was incubated in phloroglucinol solution according to Mitra and Loqué [26] and imaged.

#### Light microscopy

For morphological and histological analysis of seed retention in fodder grasses, florets of different developmental stages (before earing, earing, flowering stage, milk stage, dough stage, maturity) of contrasting genotypes of *Lolium perenne* were studied (seed retention of plants in 2018: FR 2955 (94.02%) and GR 3512 (97.42%), FR 2982 (0.72%) and GUMP-LP00441 (4.07%)). Therefore, the transition area from the rachilla to the developing caryopsis of the second floret (counted from the rachis) with a size of approx. 2 × 2 mm was cut out of a spikelet from the central area of the inflorescence (Fig. 1A-B), fixed in aldehyde, dehydrated and embedded in Spurr resin as shown in Table S1. For each stage florets of the main tiller of at least six plants were used.

**Fig. 2:**
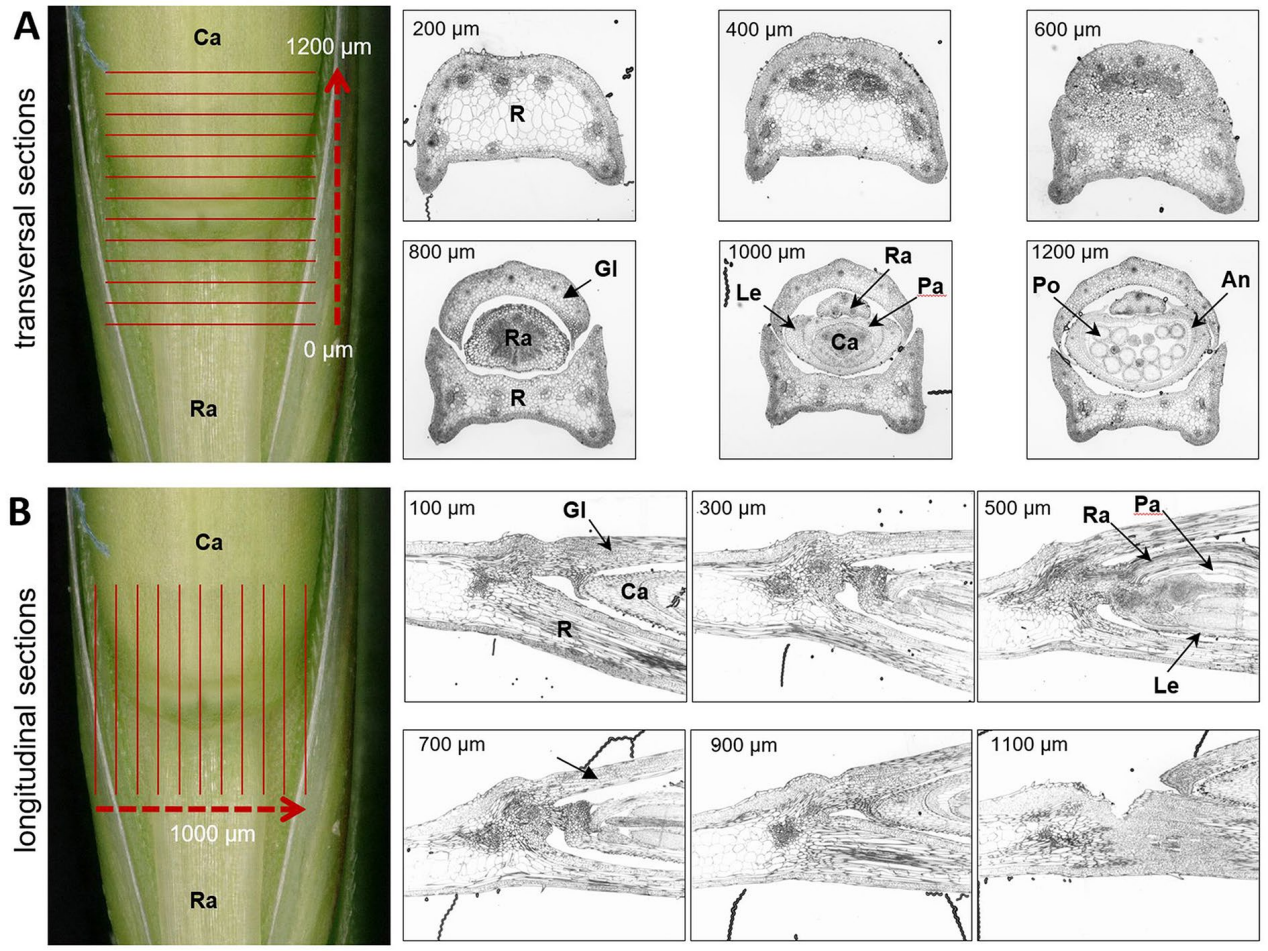
Histological section series. Resin embedded tissue of the transition zone from the rachilla to the developing caryopsis of second florets of *Lolium multiflorum* at different developmental stages were sectioned as illustrated for fresh material of earing stage (left panel of **A** and **B**). Selected sections of transversal (200–1200 μm) and longitudinal cuttings (100–1100 μm). An, anther; Ca, caryopsis; Gl, glume; Le, lemma; Pa, palea; Po, pollen; R, rachis; Ra, rachilla

To identify and characterise the zone of abscission in the different genotypes, transversal and/or longitudinal sections of 2 μm thickness were cut using a Leica UCT microtome (Leica Microsystems GmbH, Wetzlar, Germany). Sections were stained for 2 min at 60 °C with 1% (v/v) methylene blue and 1% (v/v) azur II in a 1% (v/v) aqueous borax solution. After washing, drying and permanent mounting with Entellan (Sigma-Aldrich Chemie GmbH, Taufkirchen, Germany), sections were examined with a Zeiss Axio Imager M2 light microscope equipped with a Zeiss Axiocam camera (Carl Zeiss Microscopy GmbH, Jena, Germany). Additionally, selected samples were used for serial sectioning (Fig. 2). Serial recordings were stacked and aligned using the open source Fiji/ImageJ processing software (https://imagej.net/software/fiji/downloads). For subsequent segmentation and 3D reconstruction, Amira software (version 5.6, Thermo Fisher Scientific, Germany) was used.

#### X-ray microscopy

For X-ray analysis, tissue from the transition area between the rachilla and the developing caryopsis was dissected, fixed, dehydrated and subjected to critical point drying in a Bal-Tec critical point dryer (Bal-Tec AG, Balzers, Switzerland) as described in Table S2. Dried samples were mounted on specific specimen holders and X-ray microscopy was used for multiscale high-resolution 3D imaging of the internal anatomy of the AZ.

The volumetric data of the samples shown in Figure 10 were acquired by X-ray computed tomography with the Nano CT system “ntCT”, a development of Fraunhofer EZRT [27]. It utilises a nanofocus X-ray tube with a spot size of 300 nm and a direct converting photon counting detector. In general, the system achieves a resolution of 150 nm [28] in 2D and approximately 200 nm in 3D. For the scans shown here, the system was operated in the sub-micrometer regime (0.72 μm px^− 1^) in order to visualise the entire sample. The scan time was on the order of 1 h with an exposure time of 3 s per image. Due to the ntCT’s acceleration voltage of 60 kV, organic samples show a weak attenuation contrast but a strong phase contrast, a phenomenon originating from the partial coherence of the nanofocus X-ray spot. In order to take advantage of phase contrast, i.e. improving contrast between different materials, the volumetric data was further processed with a phase retrieval algorithm based on Paganin et al. [29].

## Results

### Seed retention in 2018

**Fig. 3:**
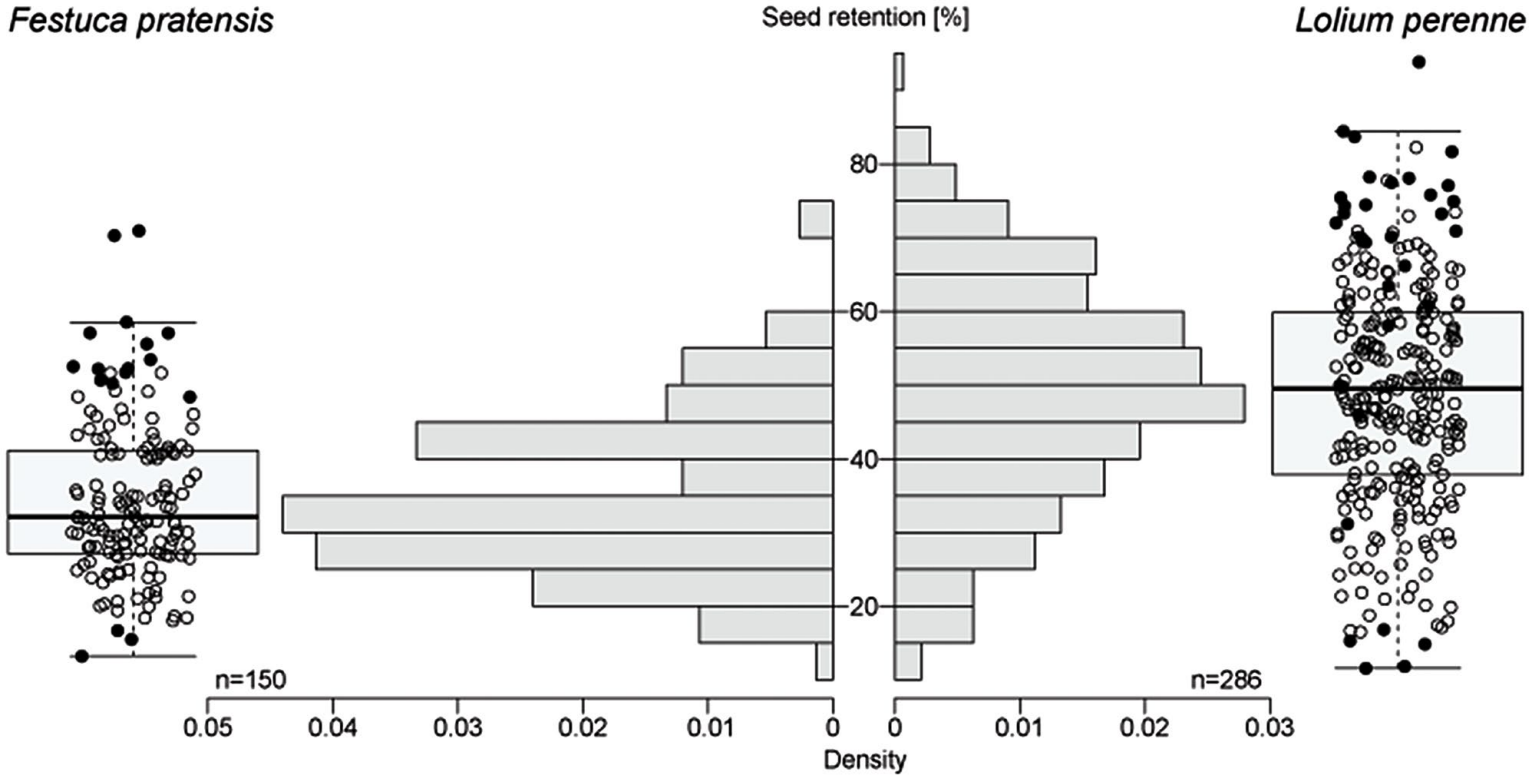
Distribution of seed retention in a diversity set of *Festuca pratensis* and *Lolium perenne* in 2018. Histogram represents the probability density of mean values per accession. Boxplot gives median, interquartile range and whiskers up to minimum and maximum values when in 1.5 times interquartile range. Displayed with a filled black circle in the overlying scatterplot are accessions that were selected for analysis in 2019. Calculation of seed retention is based on field-shattered seeds

In 2018, 150 accessions of *Festuca pratensis* and 286 accessions of *Lolium perenne* were evaluated for seed retention. Based on shattered seeds in the field, seed retention in *Festuca pratensis* ranged from 13.2 to 70.9% with a mean value of 34.5% and a median of 32.2% (Fig. 3). The two accessions with the highest seed retention were the cultivar Cosmopolitan (70.9 ± 18.0%) and the breeding population KVII03/2010 (70.3 ± 15.2%). The second cultivar GR 13010/Cosmolit and two other breeding populations tested were within the 12% of populations with highest seed retention of the set. The accession with the lowest seed retention (13.3 ± 7.0%) was the genebank accession GR 1835. The coefficient of variation ranged from 0.09 to 0.99 across all accessions with a mean value of 0.45. Seed retention correlated positively with growth related traits biomass and vigour after winter, but not with heading, seed yield per spike and rust infection (Table 1, see Additional file 1 for details). Seed weight per spike correlated positively with biomass. Heading correlated negatively with growth related traits and rust.

In *Lolium perenne*, seed retention based on shattered seeds in the field ranged from 11.6 to 93.8% with a mean value of 49.0% and a median of 49.5% (Fig. 3). The accession with the highest seed retention was genebank accession FR 2955 (93.8 ± 5.7%). The lowest seed retentions were found in the accessions GUMP-LP00441 (11.8 ± 9.5%) and ABY-Ba 8591 (11.6 ± 4.6%). Most tested cultivars and breeding populations had a seed retention lower than 50%. The cultivar with the highest seed retention in the set was Senada with 66.1 ± 21.8%. The coefficient of variation ranged from 0.06 to 0.87 across all accessions with a mean value of 0.36. Seed retention correlated significantly with all other traits: positively with heading date, negatively with vigour after winter, biomass, rust and seed weight per spike (Table 1). Heading correlated negatively with all growth traits, rust and seed weight per spike. Seed weight per spike correlated negatively with rust and positively with biomass.

**Table 1:** Correlation matrix for seed retention and other traits in *Festuca pratensis* (left lower triangle) and *Lolium perenne* (right upper triangle) in 2018

Festuca	Lolium					
	Yield per spike [g]	Rust [1–9]	Biomass [1–9]	Vigour after winter [1–9]	Heading [days]	Seed retention [%]
Yield per spike [g]		-0.215***	0.134*	-0.073^ns^	-0.260***	-0.223***
Rust [1–9]	-0.047^ns^		0.030^ns^	0.057^ns^	-0.283***	-0.258***
Biomass [1–9]	0.221**	0.151^ns^		0.666***	-0.204***	-0.376***
Vigour after winter [1–9]	0.120^ns^	0.144^ns^	0.710***		-0.210***	-0.321***
Heading [days]	0.051^ns^	-0.345***	-0.464***	-0.246**		0.638***
Seed retention [%]	0.033^ns^	-0.124^ns^	0.260**	0.247**	0.150^ns^	

Given is the Pearson’s correlation coefficient with p-values of the correlation (*p* < 0.001: ***; *p* < 0.01: **; *p* < 0.05: *; *p* > 0.05: ns)

### Seed retention in *Festuca pratensis*

**Fig. 4:**
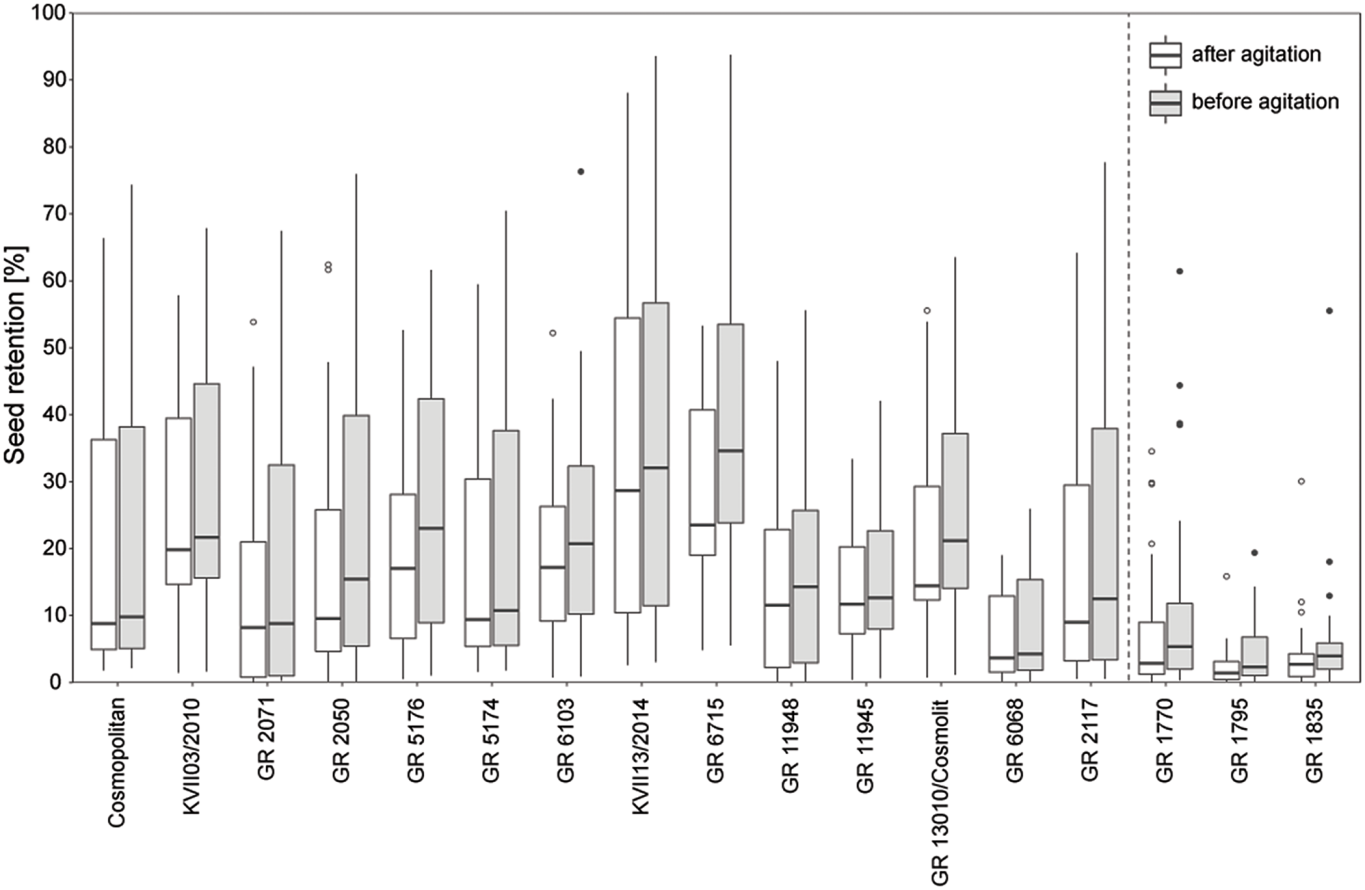
Seed retention of *Festuca pratensis* accessions in 2019 before and after agitation. Order based on seed retention in 2018 (*n* = 22–29). Boxes represent first and third quartile with a line for the median. Whiskers extend to minimum and maximum values within the 1.5 times interquartile range. Dots display outliers. Vertical dashed line separates accessions from the two groups with different seed retention level

For the analysis of seed retention of *Festuca pratensis* in 2019, 14 accessions with high seed retention and three accessions with very low seed retention in 2018 were selected (Fig. 3). The seed retention was generally lower in 2019 than in 2018 (average before agitation 2019: 21.9%; 2018: 48.5%, based on individuals analysed in both years; Fig. S3). Populations with low seed retention in 2018 remained the ones with lowest seed retention in 2019, but the difference to the populations with high seed retention in 2018 decreased (Fig. 4). The cultivar Cosmopolitan, which had the highest seed retention in 2018, exhibited only moderate retention in 2019. Agitation of spikes after harvest resulted in additional shedding of 9.4 to 40.0% of the still attached seeds, based on population means. All populations showed a high variation in seed retention. For populations with low seed retention, this variation was lower but more outliers were present. In all populations, individual genotypes were present with a seed retention before agitation below 10% and above 50%, except GR 11945 and GR 1795 with lower seed retentions. Highest variability in seed retention in the field were found for the breeding population KVII13/2014 and GR 6715 (Fig. 4).

**Fig. 5:**
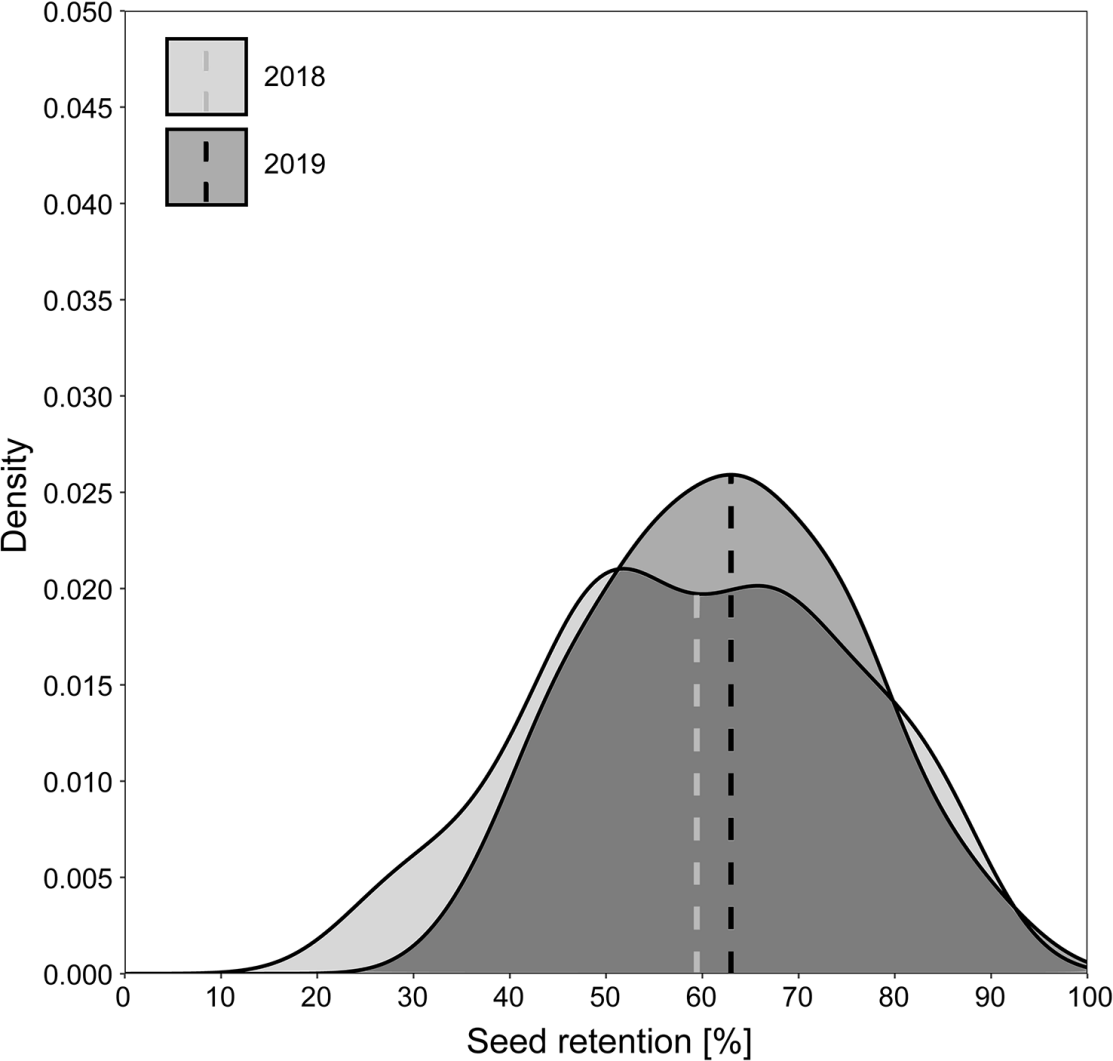
Distribution of seed retention of an F_1_ population of *Festuca pratensis* in 2018 and 2019 (with median; *n* = 79)

We additionally tested an F_1_ generation of two *Festuca pratensis* parents each with high and low seed retention in two consecutive years in Steinach. The distribution of seed retention was similar in both years with a median of around 60% seed retention after agitation, ranging from 24.4 to 87.0% in 2018 and from 34.8 to 93.3% in 2019 (Fig. 5). Hence, this population had a higher seed retention than the natural populations tested in Malchow in 2018 and 2019. Seed retention correlated over both years with a Pearson’s correlation coefficient of 0.50 (*p* < 0.001).

### Seed retention in *Lolium perenne*

For the analysis in 2019, 32 accessions of *Lolium perenne* were selected: five accessions with very low seed retention in 2018, seven accessions, mostly cultivars, with medium seed retention, and 20 accessions with high seed retention (Fig. 3).

**Fig. 6:**
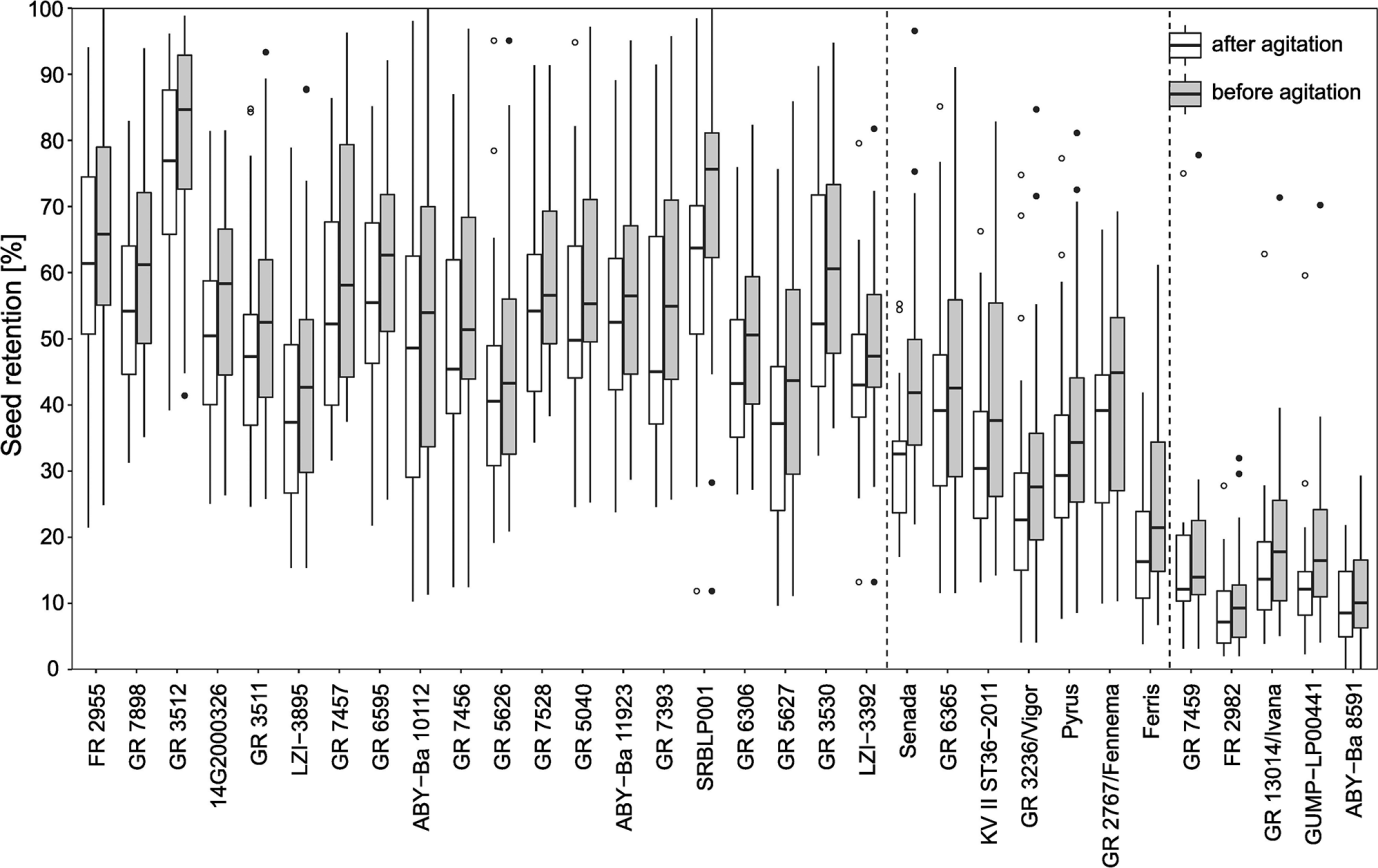
Seed retention of *Lolium perenne* accessions in 2019 before and after agitation. Order based on seed retention in 2018 (*n* = 22–30). Boxes represent first and third quartile with a line for the median. Whiskers extend to minimum and maximum values within the 1.5 times interquartile range. Dots display outliers. Vertical dashed lines separate accessions from the three groups with different seed retention level

As in *Festuca pratensis*, seed retention was generally lower in 2019 than in 2018 (average before agitation 2019: 47.4%; 2018: 61.3%, based on individuals analysed in both years; Fig. S3) and the accessions with lowest seed retention in 2018 remained the ones with lowest seed retention in 2019 (Fig. 6). The accession FR 2955 with the by far highest seed retention in 2018 was displaced by GR 3512 in 2019. Agitation after harvest resulted in detachment of 6.6 to 28.0% of the still attached seeds. Variability within the populations was high, highest in the genebank accession ABY-Ba 10112 with 11.3–100.0% seed retention in the field. Lowest seed retention variability in the field was found for ABY-Ba 8591 and FR 2982, which were the accessions with lowest seed retention in 2018 and 2019. The variability was similar in cultivars and genebank material (Fig. 6).

### Thousand grain weight of *Lolium perenne*

**Fig. 7:**
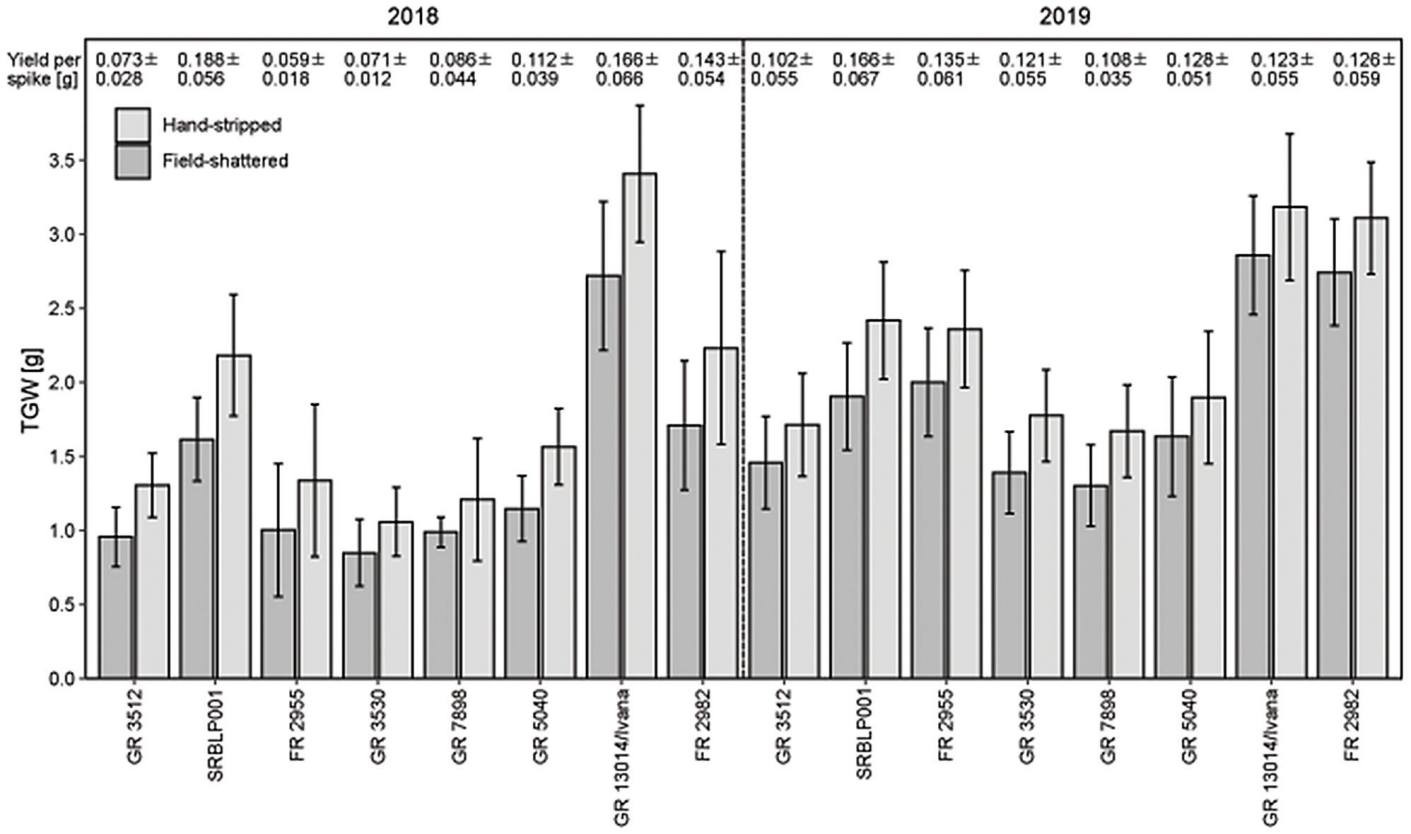
Thousand grain weight and yield per spike of *Lolium perenne.* Seeds from field-shattered and hand-stripped fractions of accessions in 2018 and 2019. Mean ± SD (*n* = 5 in 2018 and *n* = 15 in 2019)

Thousand grain weight (TGW) was determined for eight accessions grown in Malchow in 2018 and 2019. TGW was highly dependent on accession (*p* < 0.001) and overall higher in 2019 than in 2018 (*p* < 0.001, interaction significant with *p* < 0.001; Fig. 7). The field-shattered seeds had a lower TGW than the hand-stripped seeds in all accessions by on average 23.4% in 2018 and 16.8% in 2019 (*p* < 0.001 for both years). Highest TGW was found for GR 13014/Ivana with 3.0 g in 2018 and 3.1 g in 2019 (average of both fractions). Lowest TGW was found for GR 3530 in 2018 (0.9 g for field-shattered seeds, 1.2 g for hand-stripped seeds) and GR 7898 in 2019 (1.3 g for field-shattered seeds, 1.7 g for hand-stripped seeds).

Highest yield per spike was found for SRBLP001 in both years with 0.188 g in 2018 and 0.166 g in 2019. Lowest spike yield in 2018 was found for FR 2955 with 0.059 g in 2018 and for GR 3512 with 0.102 g in 2019. Spike yields were more similar between the accessions in 2019 (Fig. 7).

### Microscopy

#### Digital microscopy

**Fig. 8:**
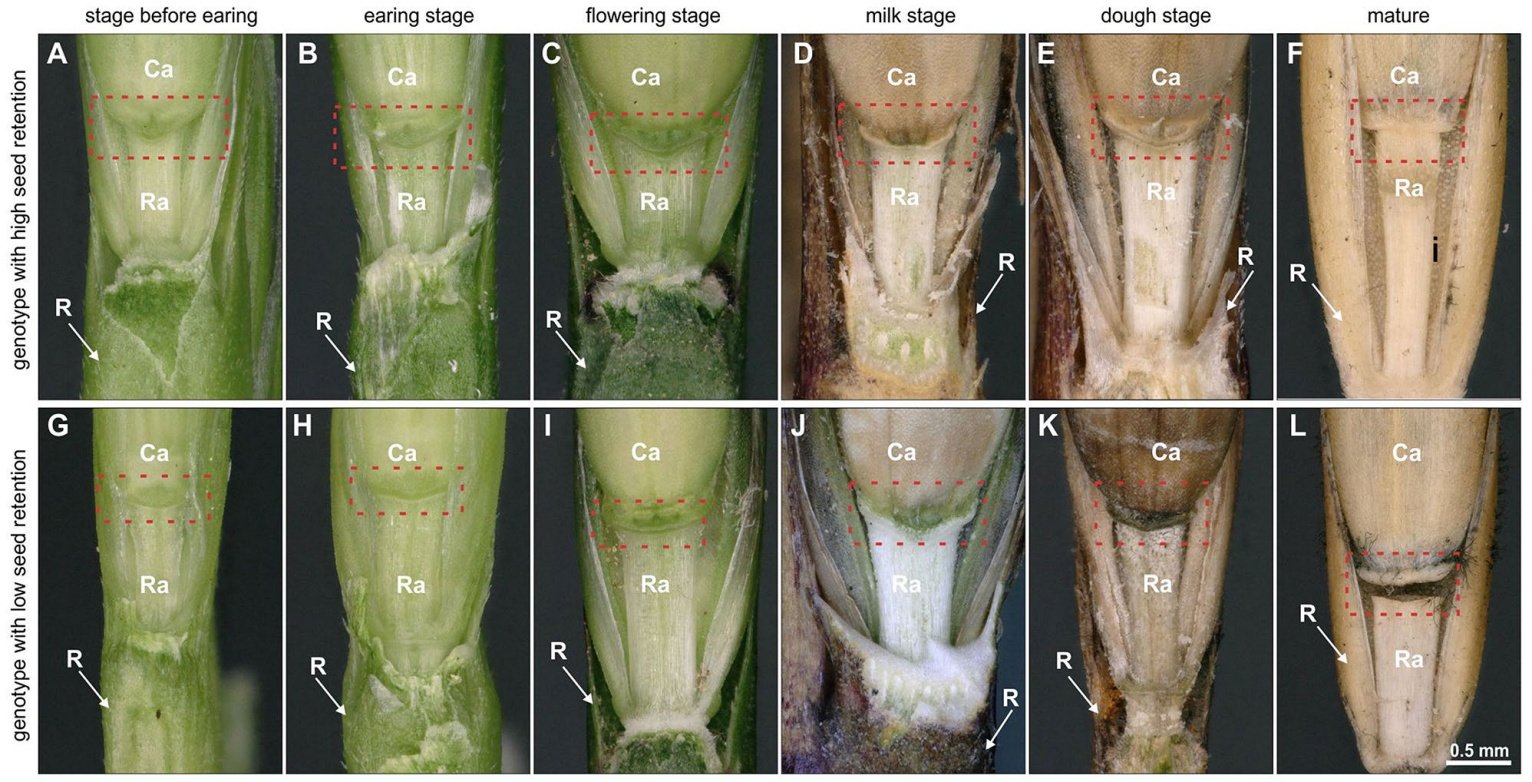
Transition zone of developing inflorescences of *Lolium multiflorum*. Digital microscopy images of genotypes with high (**A-F**) and low seed retention (**G-L**). Red boxes indicate the transition zone from the rachilla to the developing caryopsis with the putative abscission zone. Glumes of the florets were dissected. Ca, caryopsis; R, rachis; Ra, rachilla

Developmental stages from earing to milk stage did not show significant morphological differences in the transition area from the rachis to the developing caryopsis as shown for *Lolium multiflorum* (Fig. 8A-D, G-J). In dough stage, the transition area of genotypes with low seed retention appears darker (Fig. 8E, K) while mature inflorescences often showed a break in the transition zone (Fig. 8F, L). Lignin staining with phloroglucinol (Wiesner test) showed an increased degree of lignification up to the dough stage. However, no major differences were found between the examined genotypes of *Lolium perenne*, *Lolium multiflorum* and *Festuca pratensis* with low and high seed retention as shown for milk and dough stage (Supplemental Fig. S6).

#### Histology

To characterise the anatomy of the AZ as the site of disarticulation, the second florets of contrasting genotypes of *Festuca pratensis* and *Lolium perenne* at different developmental stages were used for histological studies.

**Fig. 9:**
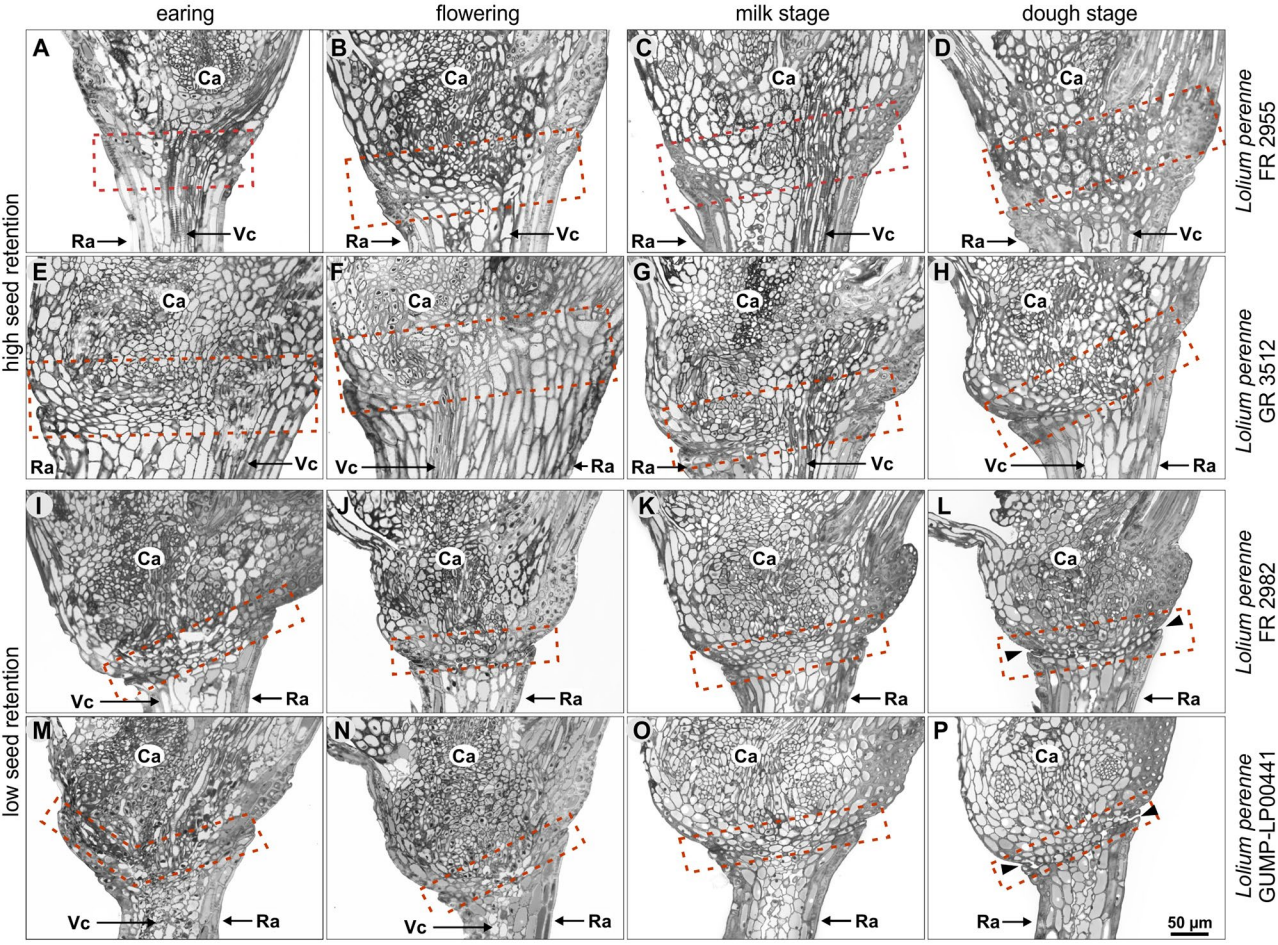
Histological characterisation of *Lolium perenne* at different developmental stages. Light microscopy images of the transition area from the rachilla to the developing caryopsis of accessions FR 2955 (**A-D**) and GR 3512 (**E-H**) with high seed retention and accessions FR 2982 (**I-L**) and GUMP-LP00441 (**I-P**) with low seed retention. Red box indicates the abscission zone and arrowheads indicate a fracture surface on the rachilla. Ca, caryopsis; Ra, rachilla; Vc, vasculature

Light microscopy analysis revealed that the cell layers of the AZ appear already in early stages as shown in Fig. 9. The AZ of genotypes of *Lolium perenne* with high seed retention are nested within each other (Fig. 9A-H), while the AZ of genotypes with low seed retention consists of two to three superimposed cell layers (Fig. 9I-P) with a smooth fracture surface on the rachilla more pronounced. Comparable results have been obtained for genotypes of *Festuca pratensis* with high (Supplemental Fig. S7A-H) and low seed retention (Supplemental Fig. S7I-P).

**Fig. 10:**
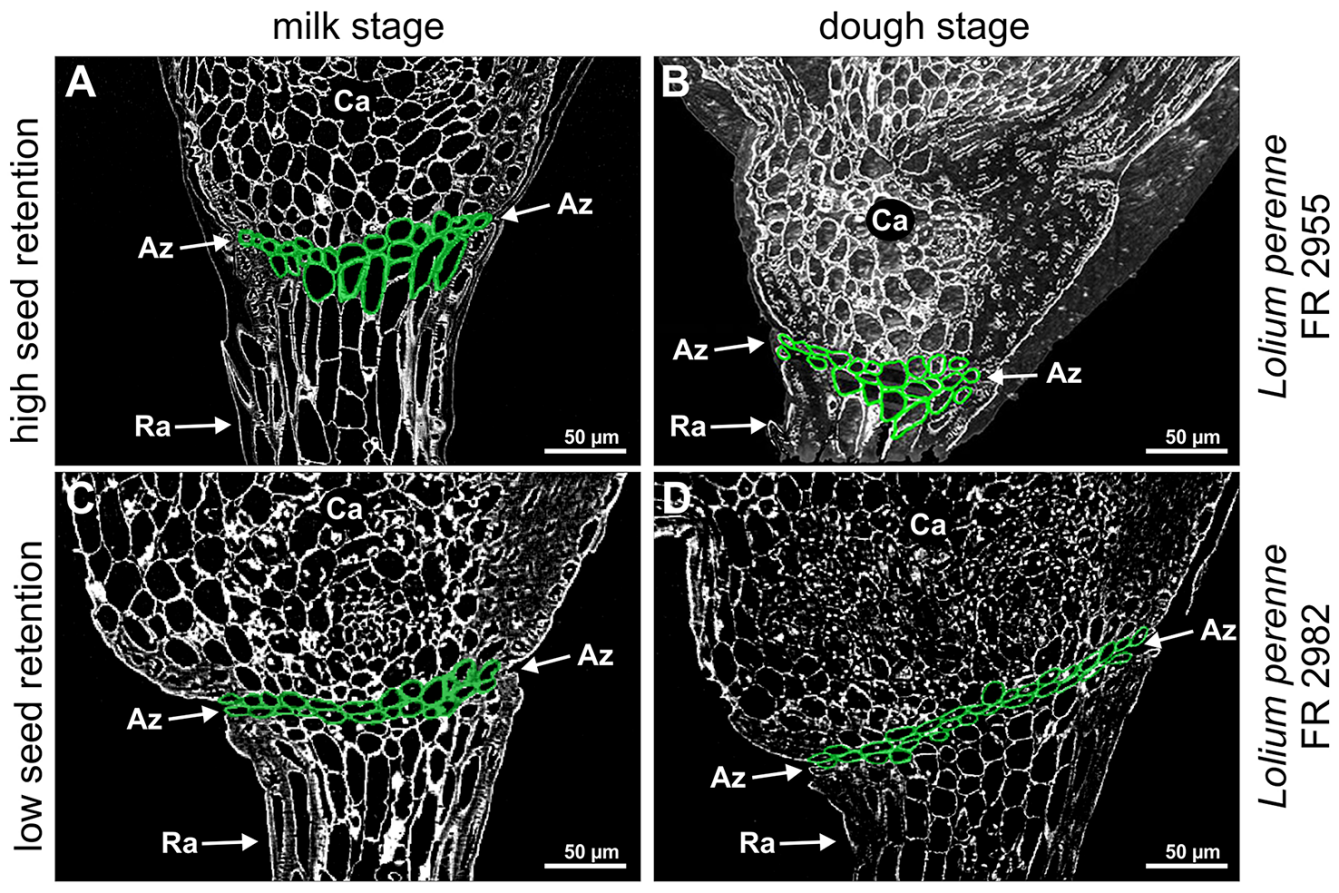
X-Ray analysis of the abscission zone of *Lolium perenne* at milk and dough stage. X-Ray analysis of tissue from milk (**A, C**) and dough stage (**B, D**) of accessions FR 2955 with high seed retention (**A-B**) and FR 2982 with low seed retention (**C-D**). Green cells indicate the abscission layer in the transition area between the rachilla and the developing caryopsis. Az, abscission zone; Ca, caryopsis; Ra, rachilla

Additionally, floret samples at milk and dough stage of contrasting accessions FR 2955 and FR 2982 were selected for X-Ray analysis and serial sectioning. X-Ray microscopy confirmed that cells of the AZ of genotypes with high seed retention are nested within each other (Fig. 10A-B, Supplemental movie S1, S2) while the AZ of genotypes with low seed retention consisted of two to three superimposed cell layers (Fig. 10C-D; Supplemental movie S3, S4). X-Ray microscopy and histological section series of the dough stage of GUMP-LP00441, an accession with low seed retention (Supplement Fig. S8; Supplemental movie S5, S6) confirmed histological data (Fig. 9M-P) and further supports X-Ray data from the accessions FR 2955 and FR 2982.

## Discussion

### Between and within population variation in seed retention

The variation in seed retention was high between and also within populations of *Festuca pratensis* and *Lolium perenne* genetic resources. Seed retention in *Festuca pratensis* was lower than in *Lolium perenne*. The inflorescence of *Lolium perenne* is a spike with the spikelets near to the rachis. *Festuca pratensis* inflorescences are awns with spikelets which are more exposed to wind. Possible is an adaption of *Festuca pratensis* to seed dispersal by wind while seed dispersal in *Lolium perenne* depends more on animals, as *Lolium perenne* plants are often lodging after flowering. Grasses with poor seed retention may lose more seeds without lodging due to wind and rain [30]. However, this hypothesis has to be tested.

A high variation within populations was expected as the genetic variation within populations is much higher than the variation between populations [3]. However, tested cultivars had also a high variation in seed retention. To test whether the high variation within the populations is due to a wide genetic background, we also tested the offspring of a cross between two single plants of *Festuca pratensis*. This family had a similar variation as the genetic resources and also the tested cultivars. Seed retention might therefore be highly influenced by multiple genes or factors other than the genetic background.

Keep et al. [21] divided a collection of European *Lolium perenne* into three phenotypic groups (G1 to G3), based on several traits describing growth, phenology, morphology and biochemistry. Of our 286 accessions, 218 could be sorted into these groups. Significantly lower seed retention was found in the G1 group. The plants in G1 are mainly from wet sites and are frost and drought sensitive [21]. They had an erect growth habit with tall fertile stems, which would also be susceptible to wind like *Festuca pratensis* inflorescences. This relation would support the above theory. However, those observations could not be replicated in our experiment, because fertile stems were tied up for observations of seed shattering and additional ones cut. But, heading was earlier than in the two other groups in our experiment as well as in Keep et al. [21]. Heading date was positively correlated with seed retention in *Lolium perenne* but not in *Festuca pratensis*. However, heading date differences were very small in *Festuca pratensis*.

### Environmental effects on seed retention

Seed retention was lower in 2019 than in 2018, with a positive correlation between both years in Malchow (Fig. S3). In our field experiment, temperatures were lower in 2018 between heading and harvest (Fig. S1), which corresponds to the observation that on sites with cooler temperature during seed maturation, seed retention was higher in grassland species [31]. In Steinach, temperature in June was higher in 2019 than in 2018 (Fig. S2). However, only very small differences were found in *Festuca pratensis* seed retention between 2018 and 2019, indicating also that plant age might not have a strong effect on seed retention level. Elgersma et al. [16] found low variation between years for cultivars of *Lolium perenne* with high seed retention and high variation for cultivars with low seed retention. This could not be confirmed in our study. Dependence of seed retention on environmental factors was found before (e.g. [32]).

### Thousand grain weight

Flowering in *Lolium perenne* starts with the proximal floret within the spikelets, with the most distal floret flowering last, while seeds ripen simultaneously [33]. The seed weight decreases from the proximal to the distal seed [33]. Distal florets can be rudimentary [16] and rarely produce seeds [33]. Shattering starts with the distal seeds of spikelets [16]. Even if more proximal seeds shatter before, the more distal seeds are not retained on the plant due to the location of the AZ across the rachilla. The lower TGW of shattered seeds compared to hand-stripped seeds in *Lolium perenne* and *Lolium multiflorum* (Supplements, Fig. S5) in our study fits well into this pattern. The TGW of shattered as well as stripped seeds of populations with low seed retention was higher than that of populations with high seed retention. Slight morphological differences were observed in the AZ of contrasting accessions of *Lolium perenne*, but also the higher seed weight might have resulted in higher shattering. The negative correlation between yield per spike and seed retention over all tested *Lolium perenne* accessions in 2018 might be a further indication for this assumption. However, we did not count seeds per spike or measured TGW of all accessions. Tubbs and Chastain [20] found that higher shattering in the tested genetic resources was associated with higher seed weight and that the tested cultivars had the highest seed weight. The one tested cultivar GR 13014/Ivana in our study had the highest seed weight of all accessions that were selected for determination of TGW. In experiments with *Lolium multiflorum*, we could demonstrate that selection for high or low seed retention, respectively, indirectly resulted in low or high TGW, respectively (Supplemental Fig. S5). High seed weight is a breeding goal for grasses. Therefore, in breeding for commercial cultivars with high seed retention, a simultaneous selection for high TGW is necessary. Additionally, biomass and vigour are crucial factors for a successful cultivar. Those are negatively, however weakly, correlated with seed retention in *Lolium perenne* in our study. The weak positive correlation in *Festuca pratensis* shows that biomass and seed retention are not directly linked and a selection for both is possible.

### Abscission zone morphology

Literature contains very different views on the anatomy and cell wall structure that characterise the AZ and thus determine the degree of seed retention, especially since seed retention can also be influenced by environmental factors.

Our histological studies on accessions of *Lolium perenne* at different developmental stages show clearly that the AZ consists of a layer of two to three cell rows with smaller cells according to findings in *Arabidopsis* [34], bean [35], rice [36] and *Lolium perenne* [16]. But in contrast to those reports, we observed this only for accessions with low seed retention. In contrast, the AZ of accessions with high seed retention consists of larger and nested cells, while Elgersma et al. [16] postulated that *Lolium perenne* shows no histological differences between plants of contrasting seed retention. A correlation between the size of the vessels in the AZ and seed retention as shown for rapeseed [37] was not observed. The investigation of different developmental stages of different accessions of *Festuca pratensis* confirms the results for *Lolium perenne*. This reinforces our observation that the size and arrangement of cells of the AZ is already determined at the heading stage at latest and that most changes within the AZ, as changes of cell wall components or lignification [9], which lead to seed retention, take place from the milk stage on. In our case, no significant differences in the degree of lignification could be shown for accessions with low and high seed retention.

Although the results again indicate that seed retention in grasses does not necessarily depend on the degree of lignification, its exact influence on the abscission zones remains unclear [10, 14].

### Breeding for seed retention

The seed retention of the tested *Lolium perenne* cultivars were in the medium range of all tested accessions. For *Festuca pratensis*, the cultivars Cosmolit and Cosmopolitan, known for high seed retention, had high seed retention also compared to the tested genebank material. In the 1960s, the cultivar Steinacher Wiesenschwingel, later renamed Cosmos 11, was irradiated to induce mutation for increasing seed retention. Successful single plants were used for recurrent selection to develop the cultivar Fesko, which led to the development of Cosmolit and later, besides others, Cosmopolitan.

Different breeding strategies for enhancing seed retention in forage grasses are described in cocksfoot (orchard grass) and meadow foxtail. Through backcross breeding, Falcinelli [4] was able to significantly increase seed retention in a cocksfoot BC_2_ population, thus combining the high level of seed retention of the donor variety with the agronomic advantages of the highly shattering recipient variety. In meadow foxtail, Simon [5] describes the development of a seed-shattering resistant cultivar derived from lines created by induced mutagenesis with gamma-irradiation. This cultivar considerably improved profitability of seed production of meadow foxtail in Germany. We show that *Lolium perenne* breeding using genetic resources as donor for high seed retention might be successful when a simultaneous selection for other important traits takes place. However, currently there is no practical way to screen for seed retention before flowering. Staining of the AZ was not successful to discriminate retention tendency on a morphological basis. Future research will focus on the identification of markers and candidate genes involved in seed retention. Such informative molecular markers may provide a useful tool in breeding for seed retention [38].

## Conclusions

This study shows that there is a high variation and potential for this important trait especially in the *Lolium perenne* genebank accessions, and gain of selection is possible as shown for *Lolium multiflorum*. However, a breeding strategy which utilises natural genetic variation has to include yield forming traits like seed weight and overall yield as they might be negatively correlated with seed retention.

### Electronic supplementary material

Below is the link to the electronic supplementary material.

Supplementary Material 1

Supplementary Material 2

Supplementary Material 3

Supplementary Material 4

Supplementary Material 5

Supplementary Material 6

Supplementary Material 7

Supplementary Material 8

## Data Availability

The datasets supporting the conclusions of this article are included within the article and its additional files. Plant material from gene banks are available by ordering from the respective gene bank, e.g. using gbis.ipk-gatersleben.de for plant genetic resources of IPK’s Genebank collection.

## References

[1] Spengler RN (2020). Anthropogenic seed dispersal. Rethinking the Origins of Plant Domestication. Trends Plant Sci.

[2] Walsh MJ, Broster JC, Aves C, Powles SB (2018). Influence of crop competition and harvest weed seed control on rigid ryegrass (*Lolium rigidum*) seed retention height in wheat crop canopies. Weed Sci.

[3] Humphreys M, Feuerstein U, Vandewalle M, Baert J, Boller B, Posselt UK, Veronesi F (2010). Ryegrasses. Fodder crops and amenity grasses.

[4] Falcinelli M. Backcross breeding to increase seed retention in cocksfoot (*Dactylis glomerata* L.). Euphytica. 1991;56:133–135. 10.1007/BF00042055.

[5] Simon U (1994). Alko the first seed-shattering resistant cultivar of meadow foxtail (*Alopecurus pratensis* L). Acta Hort.

[6] Simon U. Breeding for resistance to seed-shattering in forage grasses. Proceedings of the Third International Herbage Seed Conference, Halle (Saale), Germany, June 18–23. 1995, 119–123.

[7] Bundessortenamt L. 2022; https://www.bundessortenamt.de/bsa/media/Files/BSL/bsl_futtergraeser_2022.pdf. Accessed 07 January 2024.

[8] Estornell LH, Agustí J, Merelo P, Talón M, Tadeo FR (2013). Elucidating mechanisms underlying organ abscission. Plant Sci.

[9] Yu Y, Hu H, Doust AN, Kellogg EA (2020). Divergent gene expression networks underlie morphological diversity of abscission zones in grasses. New Phytol.

[10] Yu Y, Leyva P, Tavares RL, Kellogg EA (2020). The anatomy of abscission zones is diverse among grass species. Am J Bot.

[11] Doust AN, Mauro-Herrera M, Francis AD, Shand LC (2014). Morphological diversity and genetic regulation of inflorescence abscission zones in grasses. Am J Bot.

[12] Zhao W, Xie W, Zhang J, Zhang Z (2017). Histological characteristics, cell wall hydrolytic enzymes activity and candidate genes expression associated with seed shattering of *Elymus sibiricus* accessions. Front Plant Sci.

[13] Xie W, Zhang J, Zhao X, Zhang Z, Wang Y (2017). Transcriptome profiling of *Elymus sibiricus*, an important forage grass in Qinghai-Tibet plateau, reveals novel insights into candidate genes that potentially connected to seed shattering. BMC Plant Biol.

[14] Hodge JG, Kellogg EA (2016). Abscission Zone development in *Setaria viridis* and its domesticated relative, *Setaria italica*. Am J Bot.

[15] Wójtowicz T, Zieliński A (2021). Variability of selected traits in Meadow Fescue (*Festuca pratensis* huds.) Plants with different susceptibility to seed shattering. Biology Life Sci Forum.

[16] Elgersma A, Leeuwangh JE, Wilms HJ (1988). Abscission and seed shattering in perennial ryegrass (*Lolium perenne* L). Euphytica.

[17] Fu Z, Song J, Zhao J, Jameson PE. Identification and expression of genes associated with the abscission layer controlling seed shattering in *Lolium perenne*. AoB Plants. 2019;11(1). 10.1093/aobpla/ply076. 10.1093/aobpla/ply076 PMC6343819 30697405

[18] Maity A, Lamichaney A, Joshi DC, Bajwa A, Subramanian N, Walsh M, Bagavathiannan M (2021). Seed shattering. A trait of evolutionary importance in plants. Front Plant Sci.

[19] Maity A, Singh V, Martins MB, Ferreira PJ, Smith GR, Bagavathiannan M (2021). Species identification and morphological trait diversity assessment in ryegrass (*Lolium* spp.) populations from the Texas Blackland Prairies. Weed Sci.

[20] Tubbs TB, Chastain TG (2023). Genetic variation for seed retention in accessions and genotypic lines of perennial ryegrass (*Lolium perenne* L). Crop Sci.

[21] Keep T, Sampoux J-P, Barre P, Blanco-Pastor J-L, Dehmer KJ, Durand J-L, Hegarty M, Ledauphin T, Muylle H, Roldán-Ruiz I, Ruttink T, Surault F, Willner E, Volaire F (2021). To grow or survive: which are the strategies of a perennial grass to face severe seasonal stress?. Funct Ecol.

[22] R Core Team. R: A language and environment for statistical computing. R Foundation for Statistical Computing, Vienna, Austria. 2022. URL https://www.R-project.org/.

[23] Bates D, Maechler M, Bolker B, Walker S (2015). Fitting linear mixed-effects models using lme4. J Stat Softw.

[24] Kuznetsova A, Brockhoff PB, Christensen RHB (2017). lmerTest package: tests in linear mixed effects models. J Stat Softw.

[25] Wickham H (2016). ggplot2: elegant graphics for data analysis.

[26] Mitra PP, Loqué D (2014). Histochemical staining of *Arabidopsis thaliana* secondary cell wall elements. J Vis Exp.

[27] Müller D, Graetz J, Balles A, Stier S, Hanke R, FellaC (2021). Laboratory-based nano-computed tomography and examples of its application in the field of materials research. Crystals.

[28] Graetz J, Müller D, Balles A, Fella C (2021). Lenseless X-ray nano-tomography down to 150 nm resolution: on the quantification of modulation transfer and focal spot of the lab-based ntCT system. JINST.

[29] Paganin D, Mayo SC, Gureyev TE, Miller PR, Wilkins SW (2002). Simultaneous phase and amplitude extraction from a single defocused image of a homogeneous object. J Microsc.

[30] Boelt B, Studer B, Boller B, Posselt UK, Veronesi F (2010). Breeding for grass seed yield. Fodder crops and amenity grasses.

[31] Scotton M (2018). Wild seed harvesting at Mountainous species-Rich Grassland in Calcareous Italian Alps. Rangel Ecol Manage.

[32] San Martín C, Thorne ME, Gourlie JA, Lyon DJ, Barroso J (2021). Seed retention of grass weeds at wheat harvest in the Pacific Northwest. Weed Sci.

[33] Warringa JW, Struik PC, de Visser R, Kreuzer ADH (1998). The pattern of flowering, seed set, seed growth and ripening along the ear of *Lolium perenne*. Funct Plant Biol.

[34] Patterson SE (2001). Cutting loose. Abscission and dehiscence in Arabidopsis. Plant Physiol.

[35] Brown HS, Addicott FT (1950). The anatomy of experimental leaflet abscission in *Phaseolus vulgaris*. Am J Bot.

[36] Li L-F, Olsen KM (2016). To have and to hold: selection for seed and fruit retention during crop domestication. Curr Top Dev Biol.

[37] Child RD, Summers JE, Babij J, Farrent JW, Bruce DM (2003). Increased resistance to pod shatter is associated with changes in the vascular structure in pods of a resynthesized *Brassica napus* line. J Exp Bot.

[38] Kiesbauer J, Grieder C, Studer B, Kölliker R (2023). Perspectives for reducing seed shattering in ryegrasses. Grass Forage Sci.

