# Supplementary material for: Phenotypic screening of seed retention and histological analysis of the abscission zone in Festuca pratensis and Lolium perenne
Source: BMC Plant Biol. 2024 Jun 18;24:577. doi: 10.1186/s12870-024-05231-0 (PMC11184695; doi:10.1186/s12870-024-05231-0)
Supplement: Supplementary file 8 — Supplementary Material 8 [file 12870_2024_5231_MOESM8_ESM.pdf]

Supplements

Table S1: Protocol for combined conventional and microwave-proceeded fixation, dehydration and resin embedding of the transition area from the rachis to the developing caryopsis for histological analysis. Microwave processing (steps 1-3) in a PELCO Bio Wave®34700-230 (Ted Pella, Inc., Redding CA, USA).

Process	Reagent	Power [W]	Time [sec]	Vacuum [mm Hg]
**1. Primary fixation**	2.0 % (v/v) glutaraldehyde and 2.0 % (v/v) paraformaldehyde in 0.05 M cacodylate buffer (pH 7.3)	150<br>0<br>150<br>0<br>150	60<br>60<br>60<br>60<br>60	10<br>0<br>10<br>0<br>10
		additional over night on shaker at RT		
**2. Washing**	1x 0.05 M cacodylate buffer (pH 7.3) and 2x aqua dest.	150	45	0
**3. Dehydration**	Acetone series: 30 %, 40 %, 50 %, 60 %, 70 %, 80 %, 90 %, 2x 100 % and 1x Propylenoxide.	150	45	0
	for each step of dehydration additional 5 min on shaker			
**4. Resin infiltration**	Spurr's resin in propylenoxide on shaker.	25 %<br>50 %<br>75 %<br>100 %	over night<br>4 hrs<br>4 hrs<br>over night	
**5. Polymerisation**	24 hrs at 70°C in a heating cabinet			

Table S2: Protocol for sample preparation of the transition area from the rachis to the developing caryopsis for X-Ray microscopy. Fixation to dehydration (steps 1-5) in a PELCO Bio Wave®34700-230 (Ted Pella, Inc., Redding CA, USA).

**Process**	**Reagent**	**Power [W]**	**Time [sec]**	**Vacuum [mm Hg]**
**1. Primary fixation**	2.0 % (v/v) glutaraldehyde and 2.0 % (v/v) paraformaldehyde in 0.05 M cacodylate buffer (pH 7.3)	150<br>0<br>150<br>0<br>150	60<br>60<br>60<br>60<br>60	10<br>0<br>10<br>0<br>10
		additional over night on shaker at RT		
**2. Washing**	1x 0.05 M cacodylate buffer (pH 7.3) and 2x aqua dest.	150	45	0
**3. Secondary fixation**	1 % (v/v) osmiumtetroxide in aqua dest.	0<br>80<br>0<br>80	60<br>120<br>60<br>120	15<br>15<br>15<br>15
		additional 15 min on shaker at RT		
**4. Washing**	3x aqua dest.	150	45	0
**5. Dehydration**	Ethanol series: 30 %, 40 %, 50 %, 60 %, 70 %, 80 %, 90 %, 2x 100 %	150	45	0
	for each step of dehydration additional 15 min on shaker			
**6. Critical Point Drying** of samples in a BAL-TEC CPD 030				

Climate data Malchow

Climate data (temperature, precipitation, wind velocity and maximum speed) were obtained from the IPK weather station, located about 3 km south-east of the trial location.

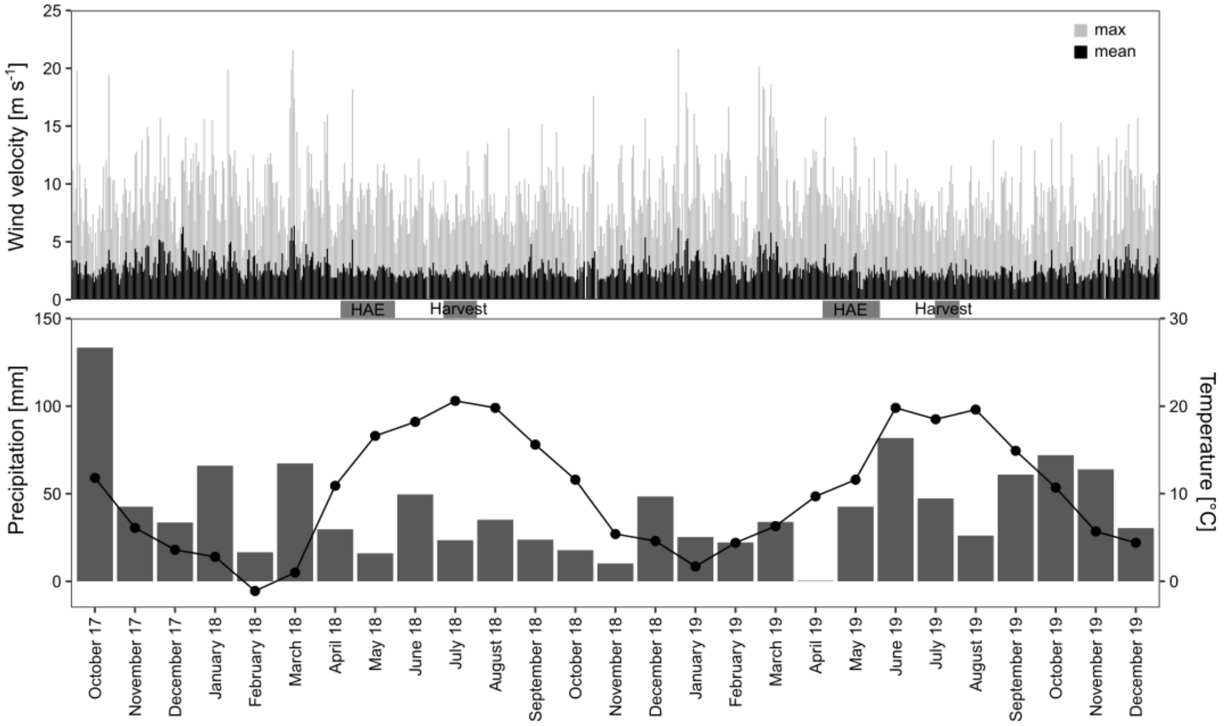

Fig. S1: Wind velocity (mean and maximum) per day, sum of monthly precipitation and temperature mean per month during the field experiment in Malchow. Period of heading (HAE) and harvest are given.

Climate data Steinach

Climate data (temperature, precipitation and wind velocity) were obtained from the weather station of the Bayerische Landesanstalt für Landwirtschaft (LfL), located about 2 km north of the trial location.

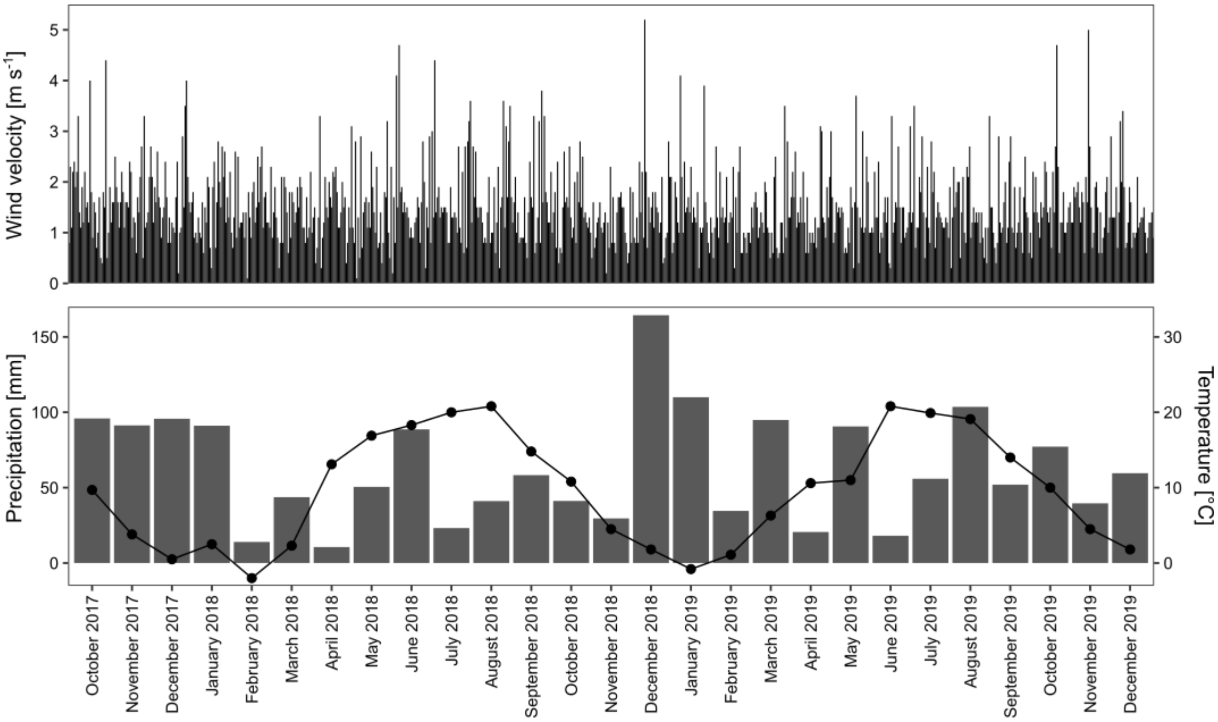

Fig. S2: Wind velocity (mean) per day, sum of monthly precipitation and temperature mean per month during the field experiments in Steinach.

Comparisons between years

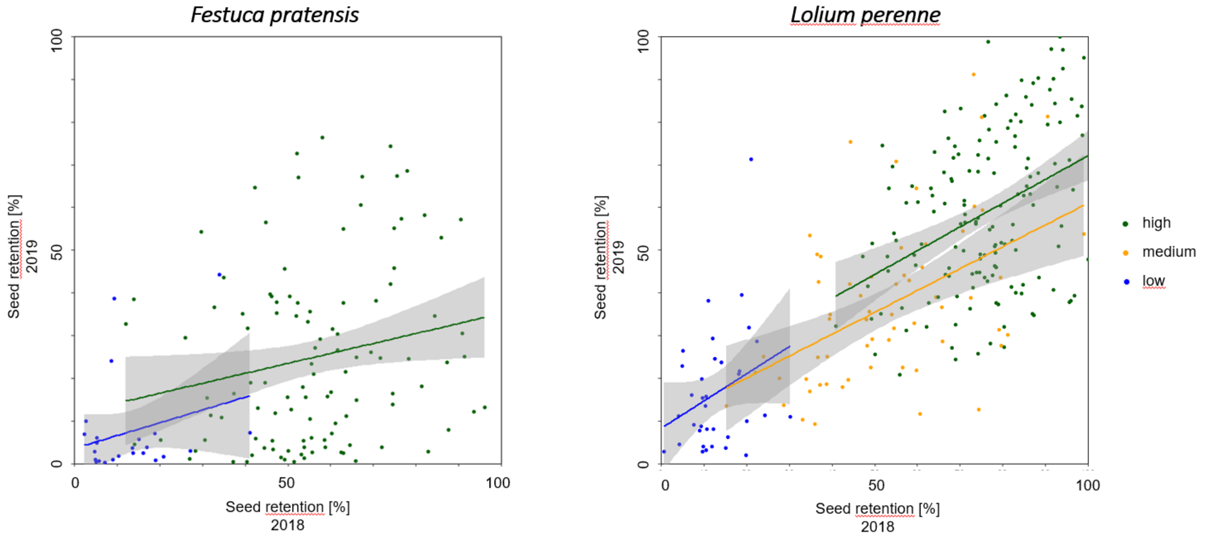

Fig. S3: Regressions between seed retentions in 2018 and 2019 in Malchow for *Festuca pratensis* and *Lolium perenne* in the groups selected for 2019 with high, medium and low seed retention.

Trial with *Lolium multiflorum* var. *westerwoldicum*

In *L. multiflorum* (diploid), the seed retention of families from two different selection processes, for high seed retention (V3) and for low seed retention (V4), was tested. Seeds from groups of plants with similar seed retention were grown and polycrossed. Progenies were tested again for seed retention and used as parents for V3 and V4 if seed retention was > 70 % or < 30 %, respectively. The first plants originate from a chemical mutagenesis experiment in order to select for genotypes with higher seed retention. *L. multiflorum* was used due to the lack of need of a vernalisation period and therefore faster breeding process. Plants were sown in 2017 in Bocksee in the north of Germany.

On average, seed retention was 76 % in V3 and 60 % in V4 before agitation. Agitation after harvest resulted in the detachment of 15 % and 22 % of the still attached seeds in V3 and V4, respectively. Variability between families was high with average seed retentions in the field between 63 % and 83 % in V3, and 45 % and 73 % in V4.

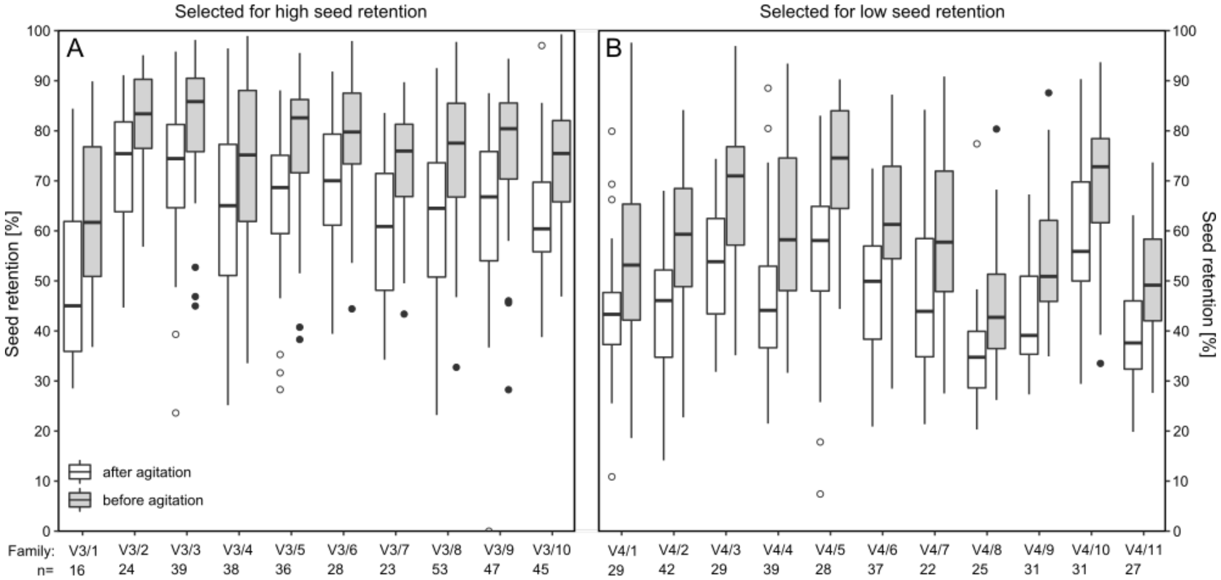

Fig. S4: Seed retention of *Lolium multiflorum* families before and after agitation (n=16-53). V3 (A) was selected for high seed retention, V4 (B) was selected for low seed retention. Boxes represent first and third quartile with a line for the median. Whiskers extend to minimum and maximum values within the 1.5 times interquartile range. Dots display outliers.

Thousand grain weight was determined for some members of the *Lolium multiflorum* families V3 and V4. TGW was on average higher in the families selected for low seed retention (V4) ($p = 0.005$; Fig. S5). The field-shattered seeds had a lower TGW than the hand-stripped seeds with seeds shattered by agitation ranging between both groups in V3 and in V4 ($p < 0.001$).

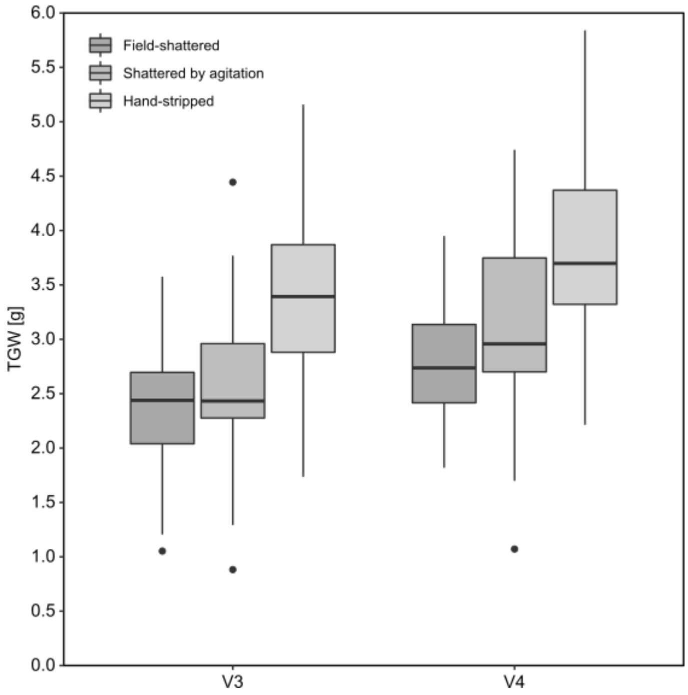

Fig. S5: Thousand grain weight of seeds from field-shattered, shattered by agitation and hand-stripped fractions of *Lolium multiflorum* families (n=53 (V3: selected for high seed retention) and n=50 (V4: selected for low seed retention)). Boxes represent first and third quartile with a line for the median. Whiskers extend to minimum and maximum values within the 1.5 times interquartile range. Dots display outliers.

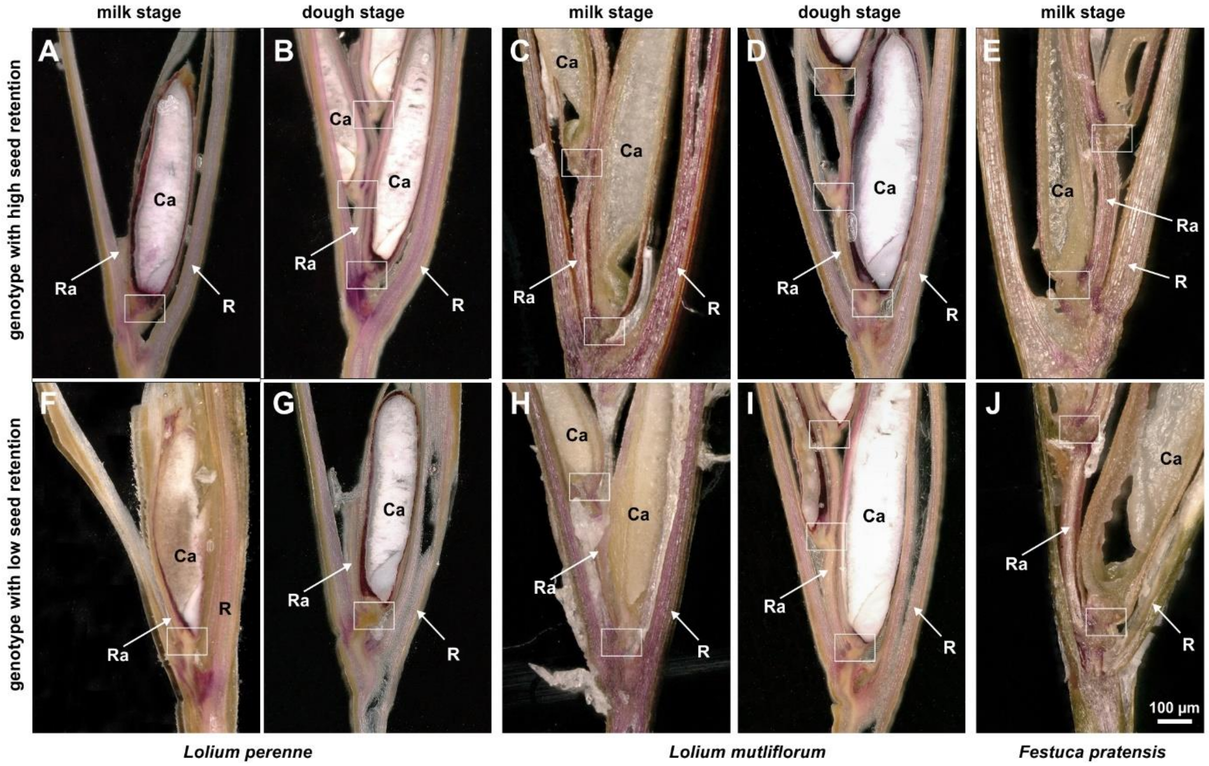

Fig. S6: Lignin staining of vibratome sections of the inflorescence of milk and dough stage. Genotypes with high (A-E) and low seed retention (F-J) of *Lolium perenne* (A-B, F-G), *Lolium multiflorum* (C-D, H-I) and *Festuca pratensis* (E, J). White box indicates the abscission zone. Ca, caryopsis; R, rachis; Ra, rachilla.

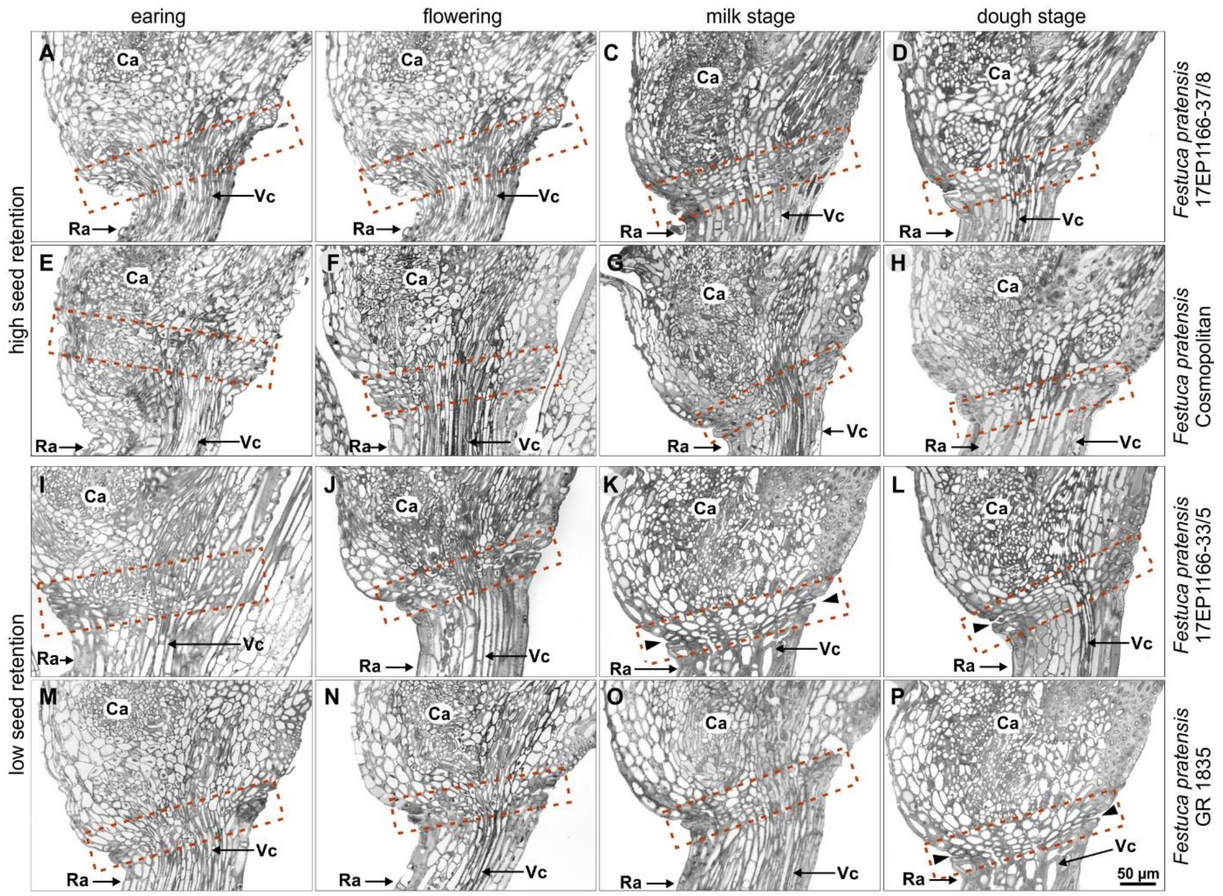

Fig. S7: Histological characterisation of the transition area from the rachilla to the developing caryopsis of *Festuca pratensis* at different developmental stages. Light microscopy images of 17EP1166-37/8 (A-D) and Cosmopolitan (E-H) with high seed retention and 17EP1166-33/5 (I-L) and GR 1835 (M-P) with low seed retention (I-P). Red box indicates the abscission zone and arrowheads indicate a fracture surface on the rachilla. Ca, caryopsis; Ra, rachilla; Vc, vasculature.

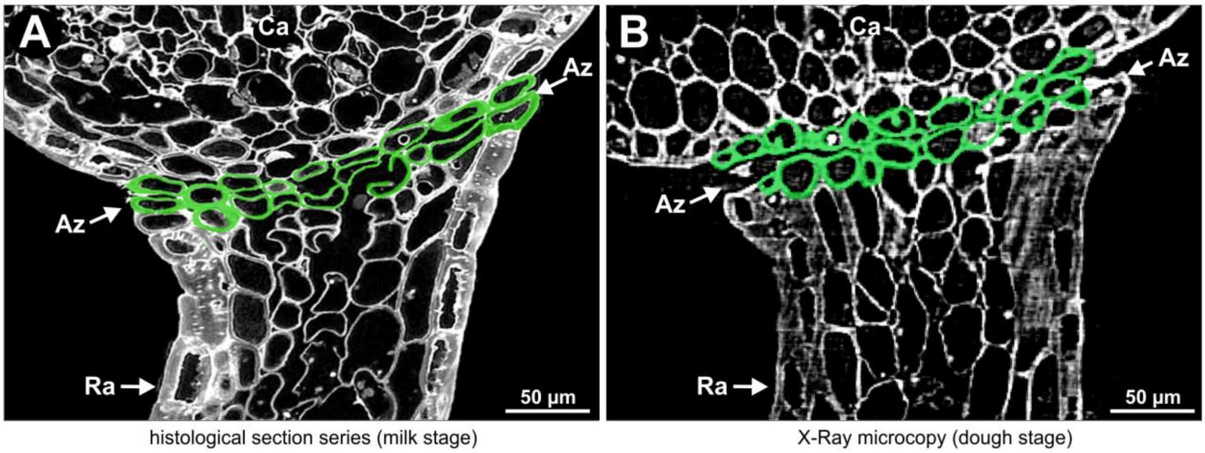

Fig. S8: Histological section series and X-Ray analysis of the abscission zone of *Lolium perenne*. Selected images of the central part of the transmission zone between the rachilla and the developing caryopsis of the accession GUMP-LP00441 with low seed retention. Green cells indicate the abscission layer after histological section series at milk stage (A) and X-Ray microscopy at dough stage (B). Az, abscission zone; Ca, caryopsis; Ra, rachilla.
